# Laboratory-scale bioaugmentation relieves acetate accumulation and stimulates methane production in stalled anaerobic digesters

**DOI:** 10.1007/s00253-015-7058-3

**Published:** 2015-10-19

**Authors:** Jennifer R. Town, Tim J. Dumonceaux

**Affiliations:** Agriculture and Agri-Food Canada, 107 Science P1, Saskatoon, SK Canada; Department of Veterinary Microbiology, WCVM, University of Saskatchewan, Saskatoon, SK Canada

**Keywords:** Anaerobic digestion, Thermophilic, Methanogen, Bioaugmentation, Acetoclastic

## Abstract

An imbalance between acidogenic and methanogenic organisms during anaerobic digestion can result in increased accumulation of volatile fatty acids, decreased reactor pH, and inhibition of methane-producing Archaea. Most commonly the result of organic input overload or poor inoculum selection, these microbiological and biochemical changes severely hamper reactor performance, and there are a few tools available to facilitate reactor recovery. A small, stable consortium capable of catabolizing acetate and producing methane was propagated in vitro and evaluated as a potential bioaugmentation tool for stimulating methanogenesis in acidified reactors. Replicate laboratory-scale batch digesters were seeded with a combination of bioethanol stillage waste and a dairy manure inoculum previously observed to result in high volatile fatty acid accumulation and reactor failure. Experimental reactors were then amended with the acetoclastic consortium, and control reactors were amended with sterile culture media. Within 7 days, bioaugmented reactors had significantly reduced acetate accumulation and the proportion of methane in the biogas increased from 0.2 ± 0 to 74.4 ± 9.9 % while control reactors showed no significant reduction in acetate accumulation or increase in methane production. Organisms from the consortium were enumerated using specific quantitative PCR assays to evaluate their growth in the experimental reactors. While the abundance of hydrogenotrophic microorganisms remained stable during the recovery period, an acetoclastic methanogen phylogenetically similar to *Methanosarcina* sp. increased more than 100-fold and is hypothesized to be the primary contributor to reactor recovery. Genomic sequencing of this organism revealed genes related to the production of methane from acetate, hydrogen, and methanol.

**Electronic supplementary material:**

The online version of this article (doi:10.1007/s00253-015-7058-3) contains supplementary material, which is available to authorized users.

## Introduction

Anaerobic digestion (AD) of agricultural waste provides an efficient disposal system for organic material while generating renewable energy in the form of methane. Thermophilic AD has been previously shown to be effective for processing stillage waste from corn- and wheat-based bioethanol production, both as a single input or co-digested with cattle manure (Agler et al. 2008; Schaefer and Sung 2008; Town et al. 2014a; Westerholm et al. 2012a). Methane generation from organic materials is reliant on the cooperative and interdependent metabolic activities of complex communities of Bacteria and Archaea, the composition and dynamics of which are in many cases poorly understood. Recent efforts to more completely and accurately characterize the microbial communities present in anaerobic digesters have resulted in a more detailed understanding of shifts in the microbial community during digestion and have identified some of the taxonomic groups responsible for each metabolic stage of methanogenesis (Vanwonterghem et al. 2014; Werner et al. 2011). The logical next step is to use this phylogenetic and taxonomic information to boost reactor performance, for example, by increasing organic loading capacity, reducing hydraulic retention time, or by altering reactor conditions to maximize the growth and metabolic capabilities of the microorganisms present in the reactor. Manipulation of the microbial community to increase reactor efficiency has also been attempted through bioaugmentation, using either complex materials like manure or compost which are rich sources of microorganisms (Schauer-Gimenez et al. 2010; Scherer and Neumann 2013; Westerholm et al. 2012a), or by the addition of exogenous cultures showing desirable metabolic properties in vitro (Akila and Chandra 2010; Fotidis et al. 2013; Westerholm et al. 2012b). The microbiological richness gained by addition of more microbially diverse input has been consistently shown to be beneficial to reactor performance, improving recovery after toxin exposure or organic overload, and greatly reducing the variability seen when processing some types of waste, including stillage (Schauer-Gimenez et al. 2010; Scherer and Neumann 2013; Westerholm et al. 2012a). Bioaugmentation with cultures of individual organisms or small syntrophic consortia has yielded mixed results, and most of the organisms tried so far are associated with hydrogenotrophic methanogenesis. The failure of these consortia to improve reactor performance has commonly been attributed to their inability to thrive in the reactor environment and to outcompete endogenous organisms for nutrients (Fotidis et al. 2013; Westerholm et al. 2012b).

Acetoclastic Archaea, in particular from the genus *Methanosarcina*, have been previously identified as robust methane producers and have been associated with reactors that have consistently elevated levels of acetate (De Vrieze et al. 2012; Lins et al. 2014). Previous genome sequencing efforts for members of this genus have indicated a high level of metabolic diversity, including pathways for acetoclastic, hydrogenotrophic, or methanol-catabolizing methanogenesis (De Vrieze et al. 2012). *Methanosarcina* spp. have also been shown to possess genes encoding cytochrome *d* oxidase and superoxide dismutase, which are hypothesized to confer aero-tolerance (Maeder et al. 2006). These characteristics suggest that organisms from this taxonomic group would make excellent targets for bioaugmentation and maximization of methane production during anaerobic digestion in cases where organic overload or the accumulation of volatile fatty acids is a concern.

This study describes the successful deployment of an acetate-catabolizing methanogenic consortium to induce methane production in reactors exhibiting symptoms of organic overload. The effect of the bioaugmentation culture on acetate accumulation and biogas production and composition was analyzed. One consortium member, a *Methanosarcina*-like acetoclastic Archaea, which showed very robust growth after deployment was selected for genome sequencing and analysis.

## Materials and methods

### Isolation and culture of bioaugmentation consortium

Minimal media for culturing acetoclastic microorganisms contained 1.5 g/L KH_2_PO_4_, 3.35 g/L Na_2_HPO_4_, 0.5 g/LNH_4_Cl, 0.18 g/L MgCl_2_•6H_2_O, 2 g/L yeast extract, 2 g/L trisodium nitriloacetate, 0.2 g/L FeCl_3_•6H_2_O, 0.2 g/L CoCl_2_•6H_2_O, 0.1 g/L MnCl_2_•4H_2_O, 0.1 g/L ZnCl_2_, 0.01 g/L NiCl_2_•6H_2_O, 0.05 g/L CaCl_2_•2H_2_O, 0.05 g/L CuSO_4_•2H_2_O, 0.05 g/L Na_2_MoO_4_•2H_2_O, 0.15 g/L Na_2_S, and 4.1 g/L sodium acetate. Media was adjusted to pH 7.0, and 10 mL was transferred to Balch tubes fitted with a rubber stopper and secured with a crimped aluminum seal. Media was flushed with N_2_ gas and evacuated twice for 1 min each and then autoclaved. Prior to inoculation, each culture tube was bled down to 6.89 kPa. Balch tubes were inoculated with 100 μL of end product digestate from a lab-scale digester containing a mixture of thin stillage and dairy cattle manure that had been producing methane for 2 weeks prior to digestate collection. The consortium was cultivated continuously at 55 °C for 6 months prior to bioaugmentation experiments and produced consistent levels of biogas in vitro as measured with a pressure transducer fitted with a 25G needle (data not shown). The composition of the consortium was determined by amplification of the type I *cpn*60 and type II thermosome universal targets (UT) as described previously (Chaban and Hill 2012; Hill et al. 2006). Amplification products for each target were cloned into the pGEM-T Easy vector (Promega, Madison, WI, USA). TOP10 cells (Life Technologies, Carlsbad, CA, USA) were transformed with the ligated vector, and complete insert sequences were determined for 64 type I and 92 type II clones. Sequences were cleaned and trimmed using LUCY (Chou and Holmes 2001) and assembled into distinct operational taxonomic units (OTU) using CAP3 (Huang and Madan 1999).

### Experimental reactors

Thin stillage was obtained from Terra Grains (Moose Jaw, SK, Canada), a dry-grind wheat ethanol facility. Fermentative inoculum was derived by incubating cattle manure from a dairy operation at the University of Saskatchewan (Saskatoon, SK, Canada) anaerobically at 55 °C for 2 weeks and stored at 4 °C until use. Total and volatile solids for the stillage and inoculum were measured using standard NREL protocols (Supplemental Table S1), and material was stored at −20 °C until use. Inputs were mixed at a 1:1 ratio based on volatile solids content and diluted with sterile water to a total solids concentration of 5 %. The input mixture was adjusted to pH 7.1 with calcium hydroxide, and ten replicate 100-mL glass jars were seeded with 30 mL of the input mixture and fitted with silicone septae. Reactors were flushed with N_2_ at 82.74 kPa for 5 min, bled down to 6.89 kPa, and incubated at 55 °C in an anaerobic chamber with an atmosphere of 85 % N_2_, 10 % H_2_, and 5 % CO_2_, as previously described (Town et al. 2014a, b). After 4 weeks, replicate reactors were amended with either 1 mL of bioaugmentation consortium or sterile culture media (*n* = 5). Digestate samples were taken using a wide-bore pipette and stored at −80 °C until use. After sampling, reactors were flushed with N_2_ gas at 82.74 kPa for 2 min using an outlet needle before being bled down to 6.89 kPa and returned to the incubator.

### Biogas analysis

Total biogas accumulation in the reactors was measured using a pressure transducer (Sper Scientific, Scottsdale, AZ, USA) fitted with a 25G needle and converted to volumes at standard temperature and pressure. Gas samples were taken weekly and extracted using a 20-mL syringe fitted with a stopcock (Cole Parmer, Vernon Hills, IL, USA) and 25 G needle, transferred to a 5-mL evacuated, dehumidified vial, and stored at 4 °C until further analysis. Biogas composition was determined using a Varian Micro-GC (CP-2003, Agilent, Santa Clara, CA, USA) equipped with a 10-m Poraplot U column and thermal conductivity detector (TCD). Relative percentages by volume of CO_2_, H_2_, H_2_S, and CH_4_ were quantified using a 10-m molecular sieve column and TCD with injector and column temperatures of 110 and 100 °C. Normalized methane was calculated as the volume of CH_4_ as a proportion of the total volume CO_2_, H_2_, H_2_S, and CH_4._

### Acetate quantification

Total acetate in the digestate samples was measured using a colorimetric acetate quantification kit according to the manufacturer’s instructions (Sigma-Aldrich, St. Louis, MO, USA). Briefly, digestate samples were centrifuged at 14,000*g* for 5 min, and 10 μL of supernatant was diluted 1/5-1/100 in water. After adding assay buffer and reaction mix to a final volume of 100 μL, reactions were incubated for 40 min at room temperature and the absorbance at 450 nm was measured.

### Quantitative PCR

Total genomic DNA was isolated from digestate samples using a modified bead beating method described previously (Dumonceaux et al. 2006). Organisms of interest were quantified using OTU-specific quantitative PCR (qPCR) assays as previously described (Town et al. 2014b). Briefly, each 25 μL reaction included 1× EvaGreen qPCR Master Mix (Biorad, Hercules, CA, USA) and 400 nM of each primer. All primer sequences and PCR conditions are detailed in Supplemental Table S2.

### Genome sequencing of OTU795

To facilitate genome sequencing and assembly for the *Methanosarcina*-like organism associated with OTU795, a selectively enriched culture was generated by treating the consortium sequentially with ampicillin (100 mg/L), kanamycin (50 mg/L), and streptomycin (50 mg/L) for 3 weeks each. Every 3–4 days, 5 mL of media was removed and replaced with fresh antibiotic-containing minimal medium as described previously. Following the enrichment process, total genomic DNA was isolated using the Wizard Genomic DNA Extraction Kit (Promega, Madison, WI, USA). The OTU795-enriched culture was sequenced using a combination of one shotgun sequencing run using Illumina chemistry on a MiSeq sequencer and one 8-kb insert paired end run using Roche454 Titanium Chemistry on a GS Junior sequencer (Hill et al. 2014). The paired end data was assembled using Newbler (v3.0) into scaffolds, and those representing OTU795 were determined based on BLASTN analysis and GC content. Illumina reads were mapped to the assembled scaffolds using Bowtie2 (Langmead and Salzberg 2012). Mapped Illumina reads for OTU795 were assembled using SOAPdenovo2 (v2.01) (Luo et al. 2012) with kmer size 127 and map length 34. The resulting contigs were then split into 500-bp pieces with a 200-bp overlap using EMBOSS splitter, combined with the paired end reads for OTU795, and re-assembled using Newbler. The sequenced genome was annotated by the Joint Genome Institute (Walnut Creek, CA, USA) and analyzed using the IMG/er portal (Markowitz et al. 2012). The assembled genome sequence can be accessed from GenBank (accession CP011449) or the Joint Genome Institute (taxon identification 2603880174). Individual genes are identified by their JGI Gene ID numbers (http://img.jgi.doe.gov).

### Accession numbers

The GenBank accession number for the genome sequence of OTU795 is CP011449, and that of OTU1109 is LDJB01000000 . Accession numbers for other OTU (*cpn*60 UT sequences) are provided in Table 1.

**Table 1:** Frequencies of typeI *cpn*60 and typeII thermosome universal targets in a clone library generated from the bioaugmentation consortium

Type I			
Frequency	Nearest neighbor cpnDB (%Identity)	OTU^a^	Accession no.^b^
3	*Thermoanaerobacter* sp. (77 %)	OTU2886	KT377171
1	*Clostridium* sp. (77 %)	OTU2575	KT377172
8	*Clostridium* sp. (78 %)	OTU2772	KT377173
2	*Symbiobacterium* sp. (96 %)	OTU2923	KT377174
1	*Desulfotomaculum* sp. (73 %)	OTU1109	KT377175
21	*Methanolinea* sp. (79 %)	OTU2750^c^	KT377176
3	*Pelotomaculum* sp. (75 %)	OTU2850	KT377177
16	*Dethiobacter* sp. (76 %)	OTU267201	KT377178
2	*Desulfotomaculum* sp. (75 %)	OTU267204	KT377179
1	*Ethanoligenens* sp. (77 %)	OTU2884	KT377180
Type II			
Frequency	Nearest neighbor cpnDB	OTU^a^	
58	*Methanoculleus bourgensis* (β-subunit) (92 %)	OTU805^c^	KT377181
29	*Methanoculleus bourgensis* (α-subunit) (84 %)	OTU807^c^	KT377182
5	*Methanosarcina barkeri* (88 %)	OTU795	KT377183

*OTU* operational taxonomic units

^a^OTU were identified in a previous study of microbial communities associated with anaerobic digestion of wheat stillage waste (Town et al. 2014b)

^b^Accession numbers for GenBank (ncbi.nlm.nih.gov)

^c^Genomic sequencing indicated that OTU2750, 805, and 807 derive from the same organism (data not shown)

## Results

### Methanogenesis in the reactors

**Fig. 1:**
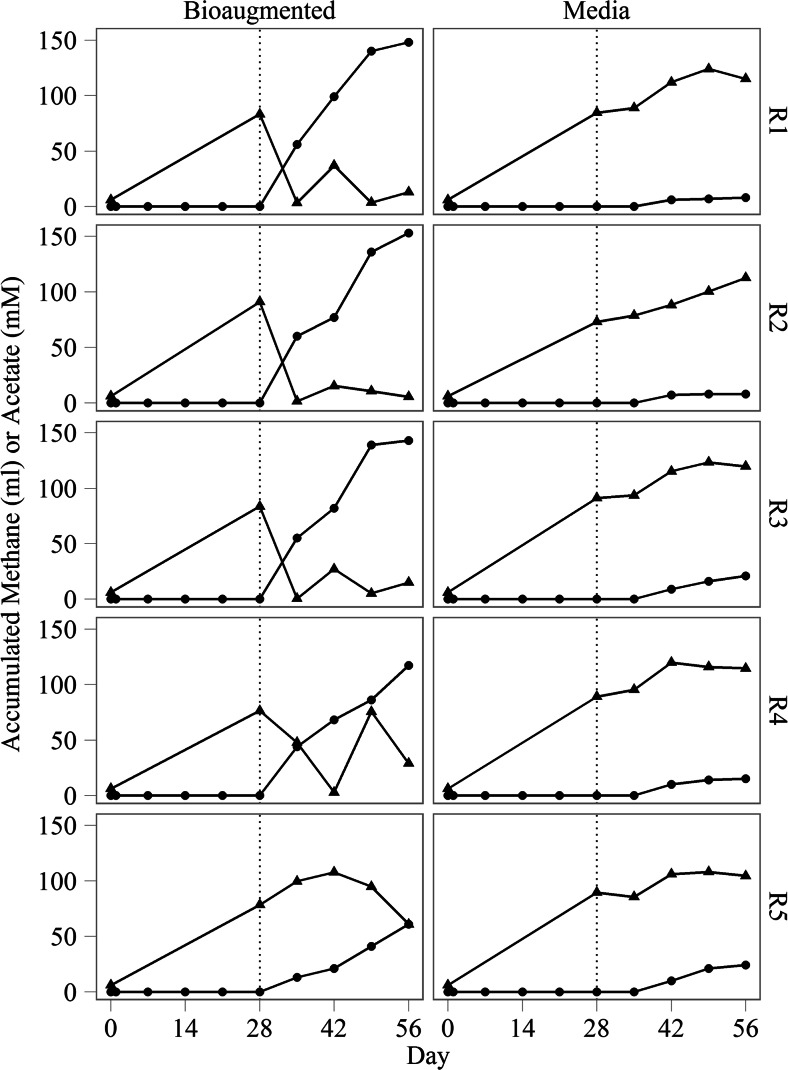
Accumulated methane (*circle*) and acetate (*triangle*) in digestate from bioaugmented and media control reactors R1–R5. Digesters were treated with either an acetoclastic consortium or sterile medium on day 28 (indicated by the *dotted line*)

**Fig. 2:**
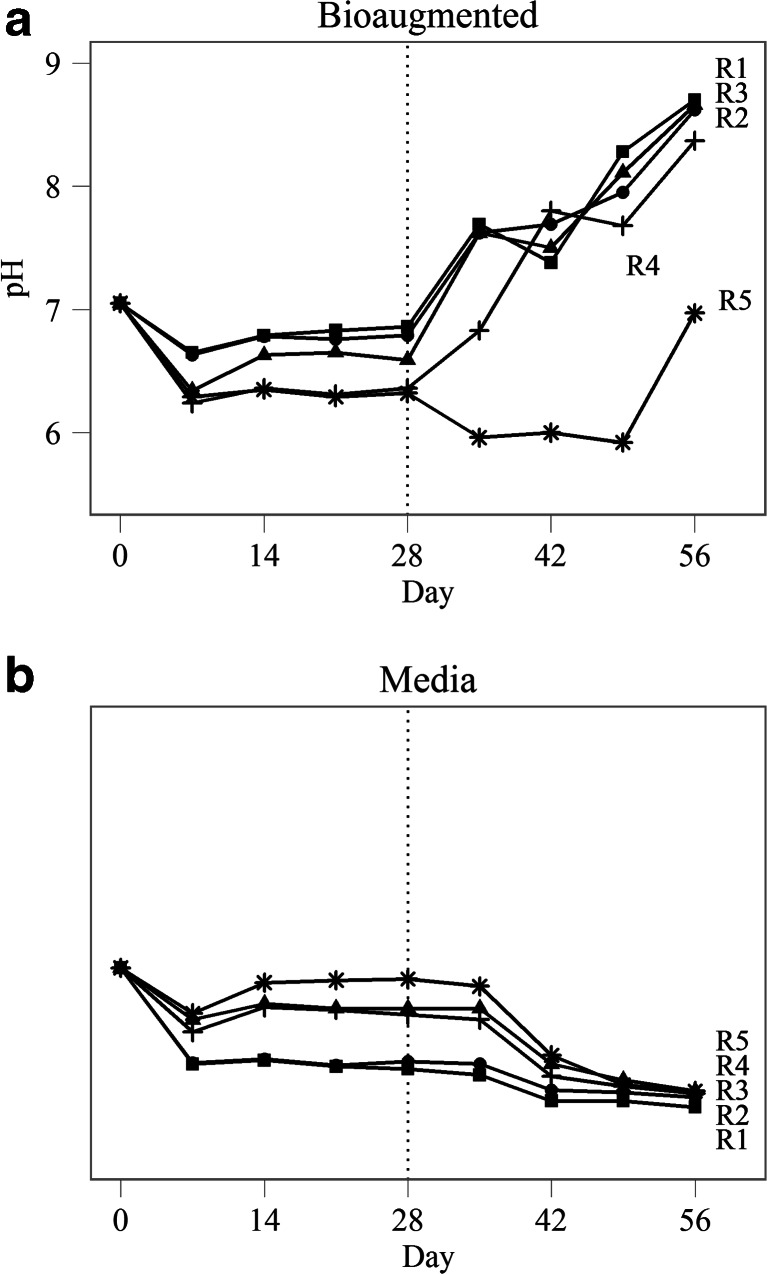
Digestate pH in bioaugmented (**a**) and media control (**b**) reactors R1–R5. Digesters were treated with either an acetoclastic consortium or sterile medium on day 28 (indicated by the *dotted line*)

The fermentative seed inoculum used in this trial had been previously shown to have abundant populations of hydrogenotrophic methanogens while acetoclastic methanogens were not detected (Town et al. 2014b). By day 28, all ten reactors showed typical characteristics of organic overload and acidification. Acetate levels increased from 6 mM at day 0 to an average of 84 ± 6.4 mM by day 28 (Fig. 1), with a corresponding decrease in pH from 7.1 to 6.6 ± 0.23 (Fig. 2). During the first 28 days of incubation, no methane was detected in the headspace of any of the reactors (Fig. 1). After addition of the acetoclastic consortium, all five bioaugmented reactors showed evidence of methanogenesis within 1 week while untreated reactors maintained signs of organic overload (Fig. 1). In four of five bioaugmented reactors, acetate levels were reduced by 62–94 % from peak levels by the end of the experimental period with a corresponding accumulation of 117–153 mL of methane, indicating that the bioaugmentation consortium was very efficient at catabolizing acetate (Fig. 1). These reactors also showed an increase in pH, which is consistent with the removal of acetate and other volatile fatty acids (Fig. 2). While there was an initial increase in acetate accumulation in reactor 5, acetate catabolization was evident by day 42, and acetate accumulation was reduced by 36 % by the end of the experimental period with 62 mL of methane produced. The lag time before onset of recovery was associated with the pH of the digestate at the time of bioaugmentation. In vitro characterization of this consortium has indicated that OTU795 grows most vigorously at pH 6.5–8 (data not shown), suggesting that the lag time before onset of reactor recovery in bioaugmented reactor R5 was a result of lower digestate pH (6.32) at the time of treatment (Fig. 2).

**Fig. 3:**
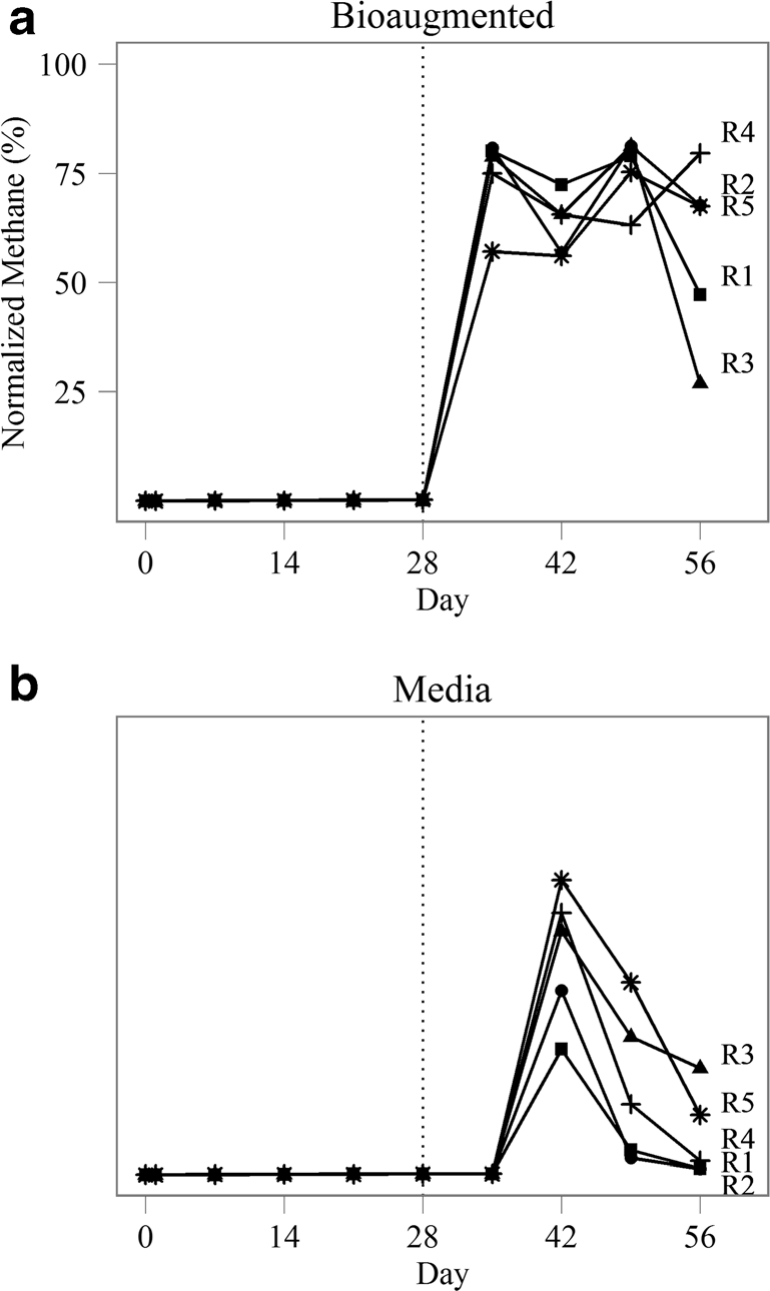
Normalized methane as a percentage of total biogas produced in bioaugmented and media control reactors R1–R5. Digesters were treated with either an acetoclastic consortium or sterile medium on day 28 (indicated by the *dotted line*)

Additionally, biogas from reactors that were amended with the acetoclastic consortium had a higher proportion of methane compared to control reactors, and this difference was maintained throughout the experimental period (Fig. 3). Unamended reactors did show some biochemical evidence of methanogenesis beginning at days 42–49 suggesting that there may have been a small increase in microbial activity stimulated by the addition of the sterile media; however, the volume of biogas produced and the relative proportion of methane were significantly lower than in the bioaugmented reactors (*p* < 0.001), and the effects were transient (Fig. 2).

### Characterization and evaluation of the bioaugmentation consortium

The bioaugmentation consortium was isolated and propagated using media and culture conditions similar to those seen in an acidified, overloaded reactor. Universal target amplification and sequencing indicated that there were ten type I (*cpn*60) and three type II (thermosome) operational taxonomic units (OTU) in the consortium representing nine bacterial and two archaeal species (Table 1). The presence of Archaea closely related to both *Methanosarcina barkeri* (OTU795) and *Methanoculleus bourgensis* (OTU805) suggested that the consortium had the metabolic capacity for both acetoclastic and hydrogenotrophic methanogenesis. The headspace of the culture vessel was not supplemented with hydrogen, suggesting that one or more of the Bacteria present in the consortium were oxidizing acetate to produce hydrogen, facilitating the growth of the hydrogenotrophic methanogen OTU805. Previous characterization of digesters processing wheat grain thin stillage identified several bacteria, including OTU1109 and OTU2923 that correlated to acetate catabolization in methane-producing thermophilic reactors (Town et al. 2014b).

**Fig. 4:**
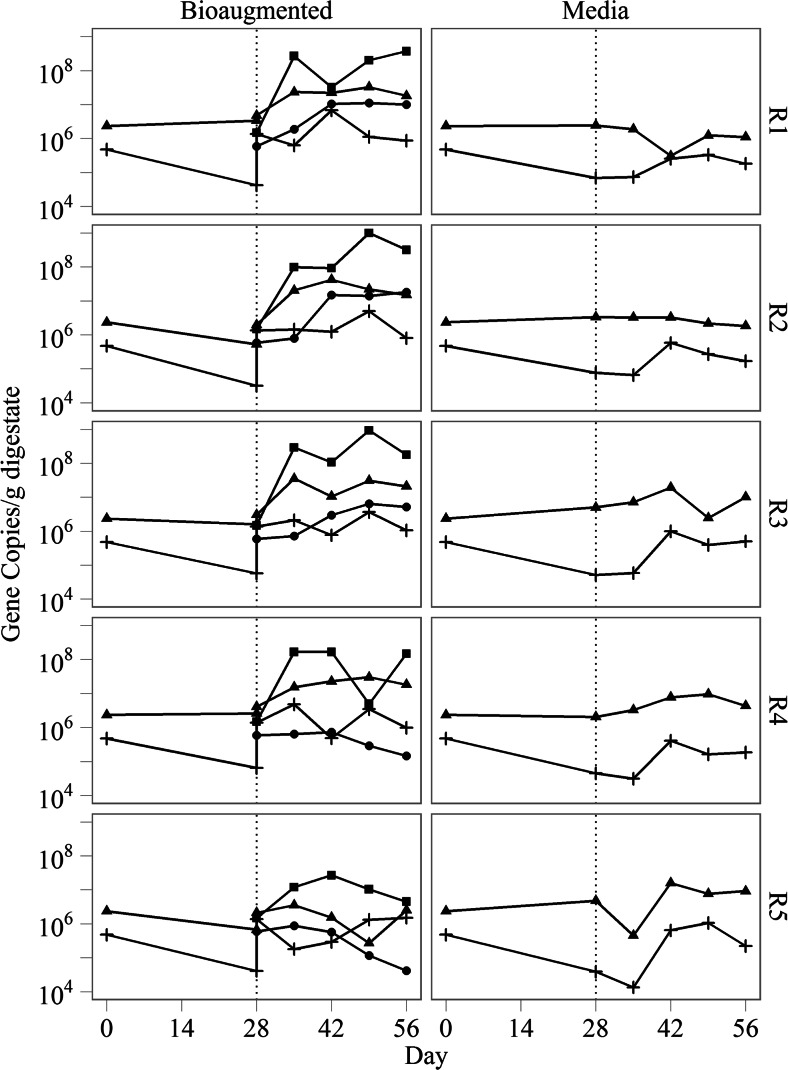
Gene abundances of bacterial *cpn*60 for OTU1109 (*circle*) and OTU2923 (*triangle*), and archaeal thermosome for OTU805 (*cross*) and OTU795 (*square*) in the digestate of bioaugmented and media control reactors R1–R5. OTU2923 and OTU795 were only detected after bioaugmentation treatment. Digesters were treated with either an acetoclastic consortium or sterile medium on day 28 (indicated by the *dotted line*)

Quantification of microorganisms from the bioaugmentation consortium using OTU-specific qPCR assays showed that while some organisms flourished, others showed no significant increase in abundance during the reactor recovery period. In particular, the acetoclastic methanogen OTU795 increased sharply during the experimental period becoming 1.33–250-fold more abundant. In contrast, the abundance of the hydrogenotrophic methanogen OTU805 as well as the hypothesized syntrophic acetate-oxidizing OTU1109 and OTU2923 remained stable during the bioaugmentation period between day 26 and day 58 (Fig. 4). Taken together, these results suggested that the primary mechanism of recovery was via acetoclastic methanogenesis from the *Methanosarcina*-like OTU795.

### Metagenomic sequencing

The draft genome for this acetoclastic Archaea consisted of one 3.1-Mb scaffold composed of nine contigs and a second 2.7-kb scaffold. A summary of general genome features is outlined in Table 2. Comparing the average nucleotide identity of OTU795 by BLAST (ANIb), MUMmer (ANIm), and tetranucleotide frequency to sequenced genomes from *Methanosarcina barkeri* Fusaro, *Methanosarcina acetivorans* C2A, *Methanosarcina mazei* Tuc01, and *Methanosarcina mazei* Go1 showed a very low identity among these strains, suggesting that this organism represents a new species within the genus *Methanosarcina* (Table 3) based on the species cut-off of 95 % identity using JSpecies (Richter and Rossello-Mora 2009). Furthermore, SpecI (Mende et al. 2013) was unable to assign OTU795 to a species cluster; *Methanosarcina barkeri* strain Fusaro (NCBI taxon ID 269797) was identified as the closest match, with an average identity of 87.1 % across 40 single copy phylogenetic marker genes. These results suggest that OTU795 represents a new or previously unsequenced species within the genus *Methanosarcina*. The ability of this organism to catabolize acetate directly likely contributed to its ability to flourish in the stalled reactors, and the ability to utilize multiple methane-producing pathways increases its potential value as an indicator of robust reactor performance.

**Table 2:** Summary of genome features for the acetoclastic methanogen represented by OTU795

	OTU795
Size (bp)	3,124,993
G + C (%)	41.21
Total genes	2757
Protein coding genes	2695
With predicted function	2084
Without predicted function	611
RNA genes	62
rRNA	6
5S	2
16S	2
23S	2
tRNA	52
Other	4

**Table 3:** Average nucleotide identity between OTU795 and several *Methanosarcina* spp. by blast (ANIb), MUMmer (ANIm), and tetranucleotide frequency

GenBank accession	JGI genome ID	Versus OTU795	ANIb	ANIm	Tetranucleotide frequency
NC_020389	2540341077	*Methanosarcina mazei* Tuc01	76.31 %	84.07 %	0.935
NC_003901	638154509	*Methanosarcina mazei* Go1	76.75 %	84.59 %	0.931
NC_007355	637000162	*Methanosarcina barkeri* Fusaro	80.53 %	85.22 %	0.927
NC_003552	638154508	*Methanosarcina acetivorans* C2A	76.73 %	84.07 %	0.911

Although substantially smaller in size at only 3.1 Mb compared to 3.4–5.8 Mb for other sequenced *Methanosarcina* spp., OTU795 appeared to have similar methanogenic capabilities. Like other *Methanosarcina* spp., the genome for OTU795 contained all genes required for methanogenic pathways originating from CO_2_, acetate, and methanol; however, it lacked the gas vesicle production (*gvp*) operon present in *Methanosarcina barkeri*. While OTU795 contained the majority of the genes required for methane production from formate, unlike *Methanosarcina barkeri*, it did not contain a complete formate dehydrogenase operon encoded by *fdhA* and *fdhB* genes (Maeder et al. 2006). Additionally, the two cytochrome *d* oxidase subunits required for oxygen respiration were present (2606038633–34), suggesting that the organism is aero-tolerant, facilitating its propagation and enhancing its resistance to occasional oxygen exposure in the digester environment. In comparing the number of genes whose predicted function fell into 1 of the 24 clusters of orthologous groups (COG) categories, it appears that the smaller genome of OTU795 has resulted in fewer genes associated with cell wall biosynthesis, and the transport and metabolism of amino acids and inorganic ions compared to other *Methanosarcina* spp. (Table 4). While it also contained fewer genes related to cell motility (Table 4), OTU795 possessed single copies of the chemotaxis genes *cheABCD* (260603938–39, 260603941–42) and multiple copies of *cheY* (2606040188, 2606040250, 2606040674, 2606040675, 2606040997) encoding a complete chemotaxis operon (Maeder et al. 2006).

**Table 4:** Summary of genome features for the acetoclastic methanogen represented by OTU795

	Number of genes				
	OTU795	*M. mazei* Tuc01	*M. mazei* Go1	*M. barkeri* Fusaro	*M. acetivorans* C2A
Genome size (Mb)	3.12	3.43	4.1	4.87	5.75
COG description					
RNA processing and modification	1	1	1	1	1
Chromatin structure and dynamics	3	3	3	3	3
Energy production and conversion	178	169	198	210	251
Cell cycle control, cell division, chromosome partitioning	15	14	14	17	16
Amino acid transport and metabolism	157	176	193	220	259
Nucleotide transport and metabolism	65	68	69	71	79
Carbohydrate transport and metabolism	74	66	79	93	107
Coenzyme transport and metabolism	154	158	176	195	243
Lipid transport and metabolism	33	27	32	35	41
Translation, ribosomal structure and biogenesis	190	190	214	218	225
Transcription	75	87	115	112	141
Replication, recombination and repair	71	64	85	80	117
Cell wall/membrane/envelope biogenesis	86	84	117	129	133
Cell motility	11	24	29	20	30
Posttranslational modification, protein turnover, chaperones	92	97	115	124	151
Inorganic ion transport and metabolism	107	121	139	207	255
Secondary metabolites biosynthesis, transport and catabolism	24	23	28	29	44

We also assembled a genomic sequence for OTU1109 from this data, which consisted of 12 scaffolds of 2.8-Mbp total length. The *cpn60* UT sequence of OTU1109 had 73 % identity to *Desulfotomaculum* sp., which placed it well below the suggested nucleotide identity for inclusion in the same species (Verbeke et al. 2011). Similar results were obtained at the whole genome sequence level, as no comparisons yielded sequence identity metrics that were able to taxonomically identify OTU1109 (Mende et al. 2013; Richter and Rossello-Mora 2009). Therefore, OTU1109 is a bacterium of unknown taxonomic lineage but which likely participates in syntrophic acetate oxidation (Town et al. 2014b).

## Discussion

Accidental overload of organic material can initiate a sequence of biochemical changes in the reactor that negatively affect methanogenesis and reduce reactor efficiency. A reactor exhibiting these symptoms is characterized by high levels of accumulated volatile fatty acids and low pH, conditions that are known to inhibit the growth of methanogenic Archaea, particularly acetoclastic methanogens (Hattori 2008). A decrease in the abundance of the acetoclastic methanogens *Methanosaeta* and *Methanosarcina* has been previously associated with increases in volatile fatty acids and decreased methane production (Carballa et al. 2015; Town et al. 2014b). Additionally, anaerobic digesters that were gradually transitioned from low to high acetate environments showed a significant increase in the abundance of *Methanosarcina* spp., suggesting that they represent a key taxonomic group for relieving volatile fatty acid accumulation during anaerobic digestion (Lins et al. 2014).

Earlier studies characterizing the microbial community during thermophilic digestion of wheat grain fermentation waste products identified individual microorganisms with abundances that correlated significantly to digester performance (Town et al. 2014a, b). In particular, the newly sequenced acetoclastic Archaea OTU795 described here had a significant positive correlation to acetate catabolization (Town et al. 2014b). This work has confirmed the causal relationship between OTU795 and the recovery of robust acetoclastic methanogenesis in acidified anaerobic digesters and suggests that *Methanosarcina* spp. show excellent potential as prospective targets for digester monitoring and bioaugmentation strategies. The use of next-generation sequencing technologies for characterizing digester communities is a valuable tool for identifying correlative relationships between microorganism abundances and digester performance, as well as novel organisms with industrial potential. In vitro characterization of these critical microorganisms can further assist in defining metabolic and growth characteristics to inform reactor operation, supplementation, and bioaugmentation strategies.

Previous attempts to improve digester performance by means of supplementation with exogenous cultures have not been as effective either because of an inability of the introduced organisms to colonize effectively (Fotidis et al. 2013), or their inability to reproduce the desired metabolic activity within a complex mixed community (Westerholm et al. 2012b). This consortium was isolated from digestate collected during a successful digestion of stillage products, increasing the likelihood that it would thrive within similar operating parameters. The use of batch digester conditions facilitated precise reactor manipulation during the acidification and recovery stages; however, it did eliminate the possibility of washout during the initial lag phase post-treatment. Washout of slow-growing exogenous cultures has been cited previously as a contributing cause of failure during reactor bioaugmentation (Fotidis et al. 2013).

An important outcome of this work is the identification of a causative, rather than correlative, relationship between the abundance of OTU795 and reactor outcomes such as decreased acetate and increased methane. A microbial consortium could conceivably be used to rescue reactors experiencing acetate accumulation along with a halt in methane production, but it is equally likely that information gained from similar studies could result in the design of reactors that provide conditions suitable for the growth of organisms that provide these functions. Deploying exogenous bioaugmentation cultures in an industrial setting is challenging, due to both larger reactor size and potential differences in reactor configuration. Identifying causal relationships between specific microorganisms and biochemical steps during methanogenesis can identify potential targets for accurate system monitoring, digestate recycling, or inoculum screening.

## Electronic supplementary material

ESM 1 (PDF 68 kb)
