# Supplementary material for: Laboratory-scale bioaugmentation relieves acetate accumulation and stimulates methane production in stalled anaerobic digesters
Source: Appl Microbiol Biotechnol. 2015 Oct 19;100:1009–17. doi: 10.1007/s00253-015-7058-3 (PMC4703610; doi:10.1007/s00253-015-7058-3)
Supplement: Supplementary file 1 — (PDF 68 kb) [file 253_2015_7058_MOESM1_ESM.pdf]

Journal: Applied Microbiology and Biotechnology

# Laboratory scale bioaugmentation relieves acetate accumulation and stimulates methane production in stalled anaerobic digesters

Jennifer R. Town[ab] and Tim J. Dumonceaux[ab]#

Agriculture and Agri-Food Canada, 107 Science Pl., Saskatoon, Saskatchewan, Canada[a]; Department of Veterinary Microbiology, WCVM, University of Saskatchewan, Saskatoon, Saskatchewan, Canada[b]

#Corresponding author. Phone +1-306-385-9450; fax +1-306-385-9482; email tim.dumonceaux@agr.gc.ca.

## Supplemental material

**Supplemental Table S1** The total (TS) and volatile (VS) solids composition of reactor input material

| | %TS | %VS (of TS) |
|---|---|---|
| Inoculum | 12.1 | 76.5 |
| Thin Stillage | 12.0 | 90.4 |
| Input | 5.02 | 81.1 |

**Supplemental Table S2** OTU-specific qPCR primer sequences and amplification conditions

| Target | Primer Sequence (5'-3') | Amplification conditions | Reference |
|---|---|---|---|
| OTU795 | CCTAAGCGTTCCCATAGAA<br>TTGCCTCTTCCTGGTCTA | 95°C 5 min (1x)<br>95°C 15 sec; 55°C 20 sec; 72°C 20 sec (40x)<br>72°C 2 min (1x) | Town et al. Bioresour. Technol. 151:249-257, 2014. |
| OTU805 | AGATCGCCATCGACATCA<br>CTCGGTGAGCTTCTCCTT | 95°C 5 min (1x)<br>95°C 15 sec; 57°C 20 sec; 72°C 20 sec (40x)<br>72°C 2 min (1x) | Town et al. Bioresour. Technol. 151:249-257, 2014 |
| OTU2923 | CCGTCCTGAACGAGCCCTA<br>GCTGCACGACCTTCTCCA | 95°C 5 min (1x)<br>95°C 15 sec; 57°C 20 sec; 72°C 20 sec (40x)<br>72°C 2 min (1x) | This study |
| OTU1109 | GTCTAACACCATCGGCACTAC<br>CGCTCGCTGTCGGTAATC | 95°C 5 min (1x)<br>95°C 15 sec; 57°C 20 sec; 72°C 20 sec (40x)<br>72°C 2 min (1x) | This study |
